# The mediation effect of placental weight change in the association between prenatal exposure to selenium and birth weight: Evidence from a prospective birth cohort study in China

**DOI:** 10.1097/EE9.0000000000000139

**Published:** 2021-04-02

**Authors:** Jiaqi Wang, Rui Qian, Yiding Wang, Moran Dong, Xin Liu, He Zhou, Yufeng Ye, Guimin Chen, Dengzhou Chen, Lixia Yuan, Jianpeng Xiao, Guanhao He, Jianxiong Hu, Weilin Zeng, Zuhua Rong, Qianqian Zhang, Mengya Zhou, Juan Jin, Jingjie Fan, Jiufeng Sun, Wenjun Ma, Bo Zhang, Tao Liu

**Affiliations:** a Guangdong Provincial Institute of Public Health, Guangdong Provincial Center for Disease Control and Prevention, Guangzhou, China; b School of Public Health, Guangdong Pharmaceutical University, Guangzhou, China; c Statistical Information Center for Health and Family Planning Bureau of Foshan, Foshan, China; d Food Safety and Health Research Center, School of Public Health, Southern Medical University, Guangzhou, China; e School of Public Health, Southern Medical University, Guangzhou, China; f Guangzhou Panyu Central Hospital, Guangzhou, China; g School of Public Health, Sun Yat-Sen University, Guangzhou, China; h Department of Prevention and Health Care, Shenzhen Maternity & Child Healthcare Hospital, Southern Medical University, Shenzhen, China; i School of Medicine, Jinan University, Guangzhou, China.

**Keywords:** Selenium, Heavy metal, Birth weight, Placental weight, Birth cohort study

## Abstract

Selenium (Se) is an essential element and also toxic at an excessive level for human body. However, few studies have investigated adverse effects of Se exposure on birth weight and placental weight.

**Methods:**

All participants were selected from the Prenatal Environment and Offspring Health cohort conducted in 2016 in Guangzhou, China. Se in each participant was measured by inductively coupled plasma mass spectrometry in their urine samples. The urinary Se concentrations were corrected by creatinine and transformed by natural logarithm (ln-Se). Multiple-linear regression models were applied to estimate the associations among Se exposure levels, placenta weight, and birth weight.

**Results:**

A total of 2758 mother-newborn pairs were included in this study. Each interquartile range (0.53 μg/g creatinine) increment in urine ln-Se concentration during the first trimester was associated with a mean 21.7 g (95% CI = −41.3g to −2.1g) decrease in birth weight and 3.6g (95% CI = −6.3g to −0.9g) decrease in placental weight. Compared with the lowest quartile (Q1) of ln-Se concentrations during the first trimester, significantly lower birth weight was found in the highest quartile (Q4) (β = −45.7g; 95% CI = −90.7g to −0.7g). Similar dose-response associations with birthweight and placental weight were found for Se exposure during the third trimester. Mediation analyses showed that 44.2% and 18.2% of the effects of Se exposure in first and third trimester on birth weight were mediated by decreased placental weight, respectively.

**Conclusion:**

Maternal Se exposure during pregnancy was negatively associated with birth weight, the reduction of placental weight may partially mediate the association of prenatal Se exposure with birth weight.

## Abstract 2

Supplemental Digital Content is available in the text.

**What this study adds**

We have conducted a birth cohort study in China and examine the association of maternal exposure to Se during pregnancy with birthweight. This study provided evidence that elevated prenatal Se exposure was negatively associated with birth weight and placental weight. We have further investigated the mediating role of placental weight change in the association between prenatal Se exposure and birth weight. This study could add more information to understand the adverse effects of Se exposure on fetal growth and development.

## Introduction

Selenium (Se) is an essential trace element in the human body,^1^ naturally presents in the earth’s crust and could enter in water and food chain via soil.^2–4^ Because the Se content in soil and water is not uniform, Se status is different by country and corresponds to intake. For example, intakes are higher in the United States, Canada, and Japan and much lower in eastern Europe.^5^ China has low Se areas from Heilongjiang province to Yunnan province; meanwhile, excessive Se intakes areas also exist in Hubei province and outfall of Yangtze river.^4,6^

Se could involve in the antioxidant and anti-inflammatory processes in the human body by forming amino acids selenocysteine, selenomethionine,^7^ and plays an important role in the maintenance of Se-dependent glutathione peroxidase (GSH-Px) activity.^8^ Previous studies have observed that the deficiency of Se was associated with heart disease, autoimmune thyroid disease, and Kaschin-Beck disease.^5,9^ However, an excessive level in the human body can lead to toxicity through inhibition of respiration and injury of gastrointestinal tract.^7^ Studies have found that a high level of Se exposure may contribute to an increased risk of type-2 diabetes.^5^ Despite the widely studied health effects of Se, there is rare study to characterize the association between maternal Se exposure during pregnancy and fetal development.^4,10^

Birth weight is an important indicator that was used to assess fetal growth and development. It is well known that the reduction of birth weight was associated with infant mortality and myriad adult health outcomes.^11^ Low birthweight (LBW), which was defined as birth weight lower than 2500 g, not only contributes to 60% to 80% of all neonatal deaths^12^ but also was associated with heart disease and type 2 diabetes.^13,14^ Heavy metal pollution was one of the risk factors, which lead to the reduction of birthweight, and many studies have implicated the association of birthweight and cadmium (Cd), thallium (Tl), and lead (Pb).^11,15,16^ However, the effects of Se on birthweight remain unclear. Thus, it is emergent and necessary to investigate the effect of prenatal Se exposure on birth weight, which could provide information for making policies to control the Se pollution and prevent the adverse effects of Se exposure on fetal health.

The potential pathway of prenatal Se exposure affecting birth weight is poorly recognized. The reduction in placental weight caused by Se exposure may play an important role.^1,10^ Placenta, a bridge between mother and fetus, is involved in the exchange of nutrients and wastes to support fetal growth.^1,11^ It was suggested that maternal high Se exposure could induce oxidative stress reaction, cause hypoxia, and disturb oxygen exchange in the placenta, which finally affects the fetal growth.^10^ Besides, the abnormal placenta was associated with gestational diabetes and preeclampsia,^17,18^ which was also associated with poorer fetal development. Several previous studies found that placental weight loss also mediates the association between birth weight and prenatal Cd and nitrogen dioxide (NO2) exposures.^16,19^ Therefore, it is plausible to hypothesize that placental weight loss may partially mediate the association between maternal exposure to Se during pregnancy with birth weight.

In this study, we conducted a Birth Cohort Study on Prenatal Environments and Offspring Health (PEOH cohort) in Guangzhou, China, beginning in 2016. We aimed to investigate the associations between maternal Se exposure during pregnancy and birth weight, further illustrate the mediation role of placental weight loss in the associations between maternal Se exposure and birth weight.

## Methods

### Study Design and Subjects

The study subjects were from the Prenatal Environments and Offspring Health (PEOH) cohort study conducted in 2016 in Guangzhou, China, which has been described elsewhere.^20–24^ Pregnant women initially recruited must meet the inclusion criteria: (a) gestational weeks ≤13 weeks; (b) aged 18–50 years; (c) no comorbidity with the following diseases: hyperthyroidism, heart disease, chronic kidney disease, tuberculosis, and psychiatric disease. The exclusion criteria were as follows: (a) the pregnant women with occupational exposure to Se-related works; (b) refuse to answer the questionnaire; (c) pregnant women have communication difficulties. The present study has obtained ethics approval from the Ethics Committee of Guangdong Provincial Center for Disease Control and Prevention and has been registered in the Chinese Clinical Trial Registry (ChiCTR-ROC-17013496). Every recruited participant was provided with a detailed introduction and explanation of this study and signed the informed consent.

Baseline and Follow-up Investigations: Upon Recruitment, the face-to-face interviews with a questionnaire were conducted, which collected information regarding maternal demographic characteristics, life behaviors, family addresses and replacement, household circumstances, diets, and history of diseases. The mean (SD) gestational week of pregnant women on the baseline investigation was 11.9 (1.5) weeks. Prenatal care records of participants were extracted from the hospital information system. All participants who completed the baseline investigation were asked to collect a 15.0 ml of spot urine, and a follow-up profile was established for them.

The follow-up investigation was conducted for each participant during their hospitalization for childbirth. The mean (SD) gestational week of pregnant women on follow-up investigation was 38.9 (1.2) weeks. All followed-up participants were required to complete the second round of the interview, and each participant was collected a 15.0 ml of spot urine. The birth record of each newborn was extracted from the maternal medical record. The follow-up investigation was finished in December 2017.

### Endpoints

Birth weight and placental weight were measured by the midwife at the time of delivery and recorded in the maternal medical record immediately. A total of 4928 pregnant women were finally recruited in the baseline investigation, and 4279 were successfully followed up (86.8%). We further excluded participants who did not donate urine samples in the baseline investigation (N = 2058), were not recorded the birth weight (N = 8) and placental weight (N = 15), multiple births (N = 31), stillbirth (N = 3), and lack of key variables such as maternal education (N = 1), family income (N = 10), and prepregnancy body mass index (BMI) (N = 2). As a result, 2151 pregnant women were included in the analysis of estimating the association of maternal Se exposure during the first trimester with birth weight (eFigure S1; http://links.lww.com/EE/A128).

In the 4279 followed-up participants, we excluded participants who did not donate urine samples (N = 2840) during their hospitalization for childbirth, with no information of birth weight (N = 26) and placental weight (N = 15), multiple births (N = 10), and loss of key variables such as maternal age (N = 1), maternal education (N = 1), and prepregnancy weight (N = 3). A total of 1383 pregnant women were finally included to estimate the association between maternal Se exposure during the third trimester with birth weight. Finally, a total of 2758 pregnant women who were included in these two subgroups were analyzed in this study (eFigure S1; http://links.lww.com/EE/A128).

### Determinations of the Urinary Concentrations of Se and Creatinine

We stored all urine samples in polypropylene tubes at –80°C after collection. All urine samples thawed at room temperature until completely melted before measurement. We have described the detailed methods of metal and creatinine concentration measurement elsewhere.^25^ Briefly, the concentrations of Se in the collected samples were measured with an inductively coupled plasma mass spectrometer (ICP-MS) (Agilent 7700x, Agilent Technologies) at the laboratory of Guangdong Provincial Center for Disease Control and Prevention.

ICP-MS measurement conditions included RF power 1550 W, plasma gas flow 15.00 L/min, carrier gas flow 1.14 L/min, helium gas flow 4.5 L/min, and so on. The limit of detection (LOD) for Se was 0.1 (μg/L). The urine samples with Se concentrations below the limit of detection were assigned a value of one-half of the LOD^25^ We employed the urinary creatinine to calibrate the urinary Se concentration. The concentrations of urinary creatinine were assessed by an automatic biochemical analyzer (Hitachi 7600-020) according to the improved Jaffe reaction with an intent to correct urine volume differences and individual differences.

### Covariates

The following variables were considered as potential confounders according to the references.^2,16,26,27^ Maternal sociodemographic characteristics (maternal age, education, and household income), maternal lifestyle factors (maternal smoking, passive smoking and alcohol consumption), maternal medical information (prepregnancy BMI, parity, gravidity, gestational weeks, infant sex, and adverse pregnant history).

### Statistical Analysis

The ANOVA analysis or *t*-tests were employed to compare the differences of the distribution of placental weight and birth weight among subgroups. The urinary Se concentrations were corrected by creatinine (CC-Se). Because the CC-Se distribution is left-skewed, a natural logarithm transformation was used to normalize the distributions of the CC-Se for statistical analyses.^4,11^ A multiple linear regression model was used to estimate the associations between maternal urinary ln-Se levels and neonatal birth weight. We reported birth weight change (β, 95% CI) and placental weight change (β, 95% CI) of the per interquartile range (IQR) of ln-Se concentration, and for the second (Q2), third (Q3), and fourth (Q4) quartile compared with the first quartile (Q1) of ln-Se concentration. The multiple linear regression model was also used to investigate the associations between placental weight and birth weight.

Furthermore, we performed a mediation analysis with a series of multiple linear regression models after adjusting potential confounders to determine whether placenta weight change is a potential mediator in the association between maternal urinary ln-Se levels and birth weight.^28^

A three-step method was used in the mediation analysis. First, we standardized maternal urinary ln-Se levels, birth weight, and placental weight of our data by its mean and standard deviation to calculate the Z-score. Second, the linear regression models were used to test the association of ln-Se Z-score, placental weight Z-score, and birth weight Z-score. Third, we calculated the proportion of mediation as 1 −(c’/c), in which c denotes the standardized coefficient of ln-Se Z-score with birth weight Z-score without adjustment for placental weight Z-score, and c’ denotes the standardized coefficient after adjustment for placental weight Z-score (eFigure S1; http://links.lww.com/EE/A128).

All analyses were performed using R3.6.1 (R Development Core Team 2019, https://www.r-project.org). All tests were two-sided, and *P* < 0.05 was considered statistically significant.

## Results

### General Characteristics of the Study Population

A total of 2758 mother-newborn pairs were finally included in the present study, and the general characteristics of participants are described in Table 1. The average birth weight of neonates and placental weight was 3193.8 g (SD = 406.7 g) and 521.3 g (SD = 52.0 g), respectively. The mean (±SD) Se exposure levels in the first trimester and the third trimester were 44.6 ± 25.0 μg/g creatinine and 46.7 ± 31.8 μg/g creatinine, respectively. After the logarithmic transformation, the mean (±SD) ln-Se exposure levels in the first trimester and the third trimester were 3.7 ± 0.4 μg/g creatinine and 3.7 ± 0.7 μg/g creatinine, respectively. The Se concentrations in participants with different characteristics were shown in eTable S1; http://links.lww.com/EE/A128. Out of the total participants, 2613 (94.7%) were aged 26 years or older, 2658 (96.4%) had term births (>37 gestational weeks), only 4 of participants had smoked during their pregnancy, and 883 (32.0%) had passive smoking exposure during pregnancy. Neonates birth weight was significantly higher among pregnant women with gestational weeks >37, having a male infant, higher gravidity, higher prepregnancy BMI and household income. Placental weight was significantly higher in pregnant women with younger age, having a male infant, and without gestational diabetes and hypertension (Table 1).

**Table 1.** Characteristics of study participants.

	N (%)	Birth weight (g)Mean ± SD	t/F	*P*	Placental weight (g) Mean ± SD	t/F	*P*
Maternal age (years)			2.37^a^	0.069		6.13^a^	<0.001
<25	145 (5.3)	3179.3 ± 411.8			533.2 ± 59.2		
26~29	919 (33.3)	3169.1 ± 394.2			524.5 ± 56.4		
30~34	967 (35.1)	3217.3 ± 400.9			520.7 ± 48.8		
≥35	727 (26.3)	3196.7 ± 427.2			515.4 ± 48.1		
Gestational age (weeks)			130.53^a^	<0.001		29.71^a^	<0.001
<37	100 (3.6)	2549.5 ± 459.2			485.7 ± 46.8		
37~41	2526 (91.6)	3207.2 ± 383.6			521.9 ± 51.3		
≥42	132 (4.8)	3426.1 ± 332.9			536.3 ± 58.4		
Infant sex			6.74^b^	<0.001		2.06^b^	0.039
Male	1492 (54.1)	3241.5 ± 401.9			523.2 ± 53.3		
Female	1266 (45.9)	3137.6 ± 405.3			519.1 ± 50.4		
Parity			6.35^a^	0.002		0.72^a^	0.487
0	957 (34.7)	3156.8 ± 408.5			522.6 ± 52.3		
1	1671 (60.6)	3215.4 ± 405.9			520.9 ± 52.1		
≥2	130 (4.7)	3188.5 ± 386.2			517.2 ± 49.3		
Gravidity			5.32^a^	0.001		0.68^a^	0.562
1	754 (27.3)	3150.3 ± 395.4			523.6 ± 54.0		
2	1106 (40.1)	3200.6 ± 409.4			520.4 ± 51.4		
3	598 (21.7)	3206.3 ± 397.0			520.1 ± 48.3		
≥4	300 (10.9)	3253.2 ± 433.9			521.7 ± 56.4		
Maternal education (years)			7.04^a^	0.03		2.01^a^	0.134
≤12	433 (15.7)	3176.1 ± 423.5			520.9 ± 52.8		
13~15	1575 (57.1)	3194.8 ± 389.5			523.6 ± 53.2		
>15	750 (27.2)	3215.5 ± 407.2			518.7 ± 49.2		
Household income (×1000 Yuan)			10.99^a^	<0.001		2.42^a^	0.066
<30	123 (4.5)	3087.8 ± 366.8			518.9 ± 45.5		
30~	1682 (61.0)	3172.1 ± 412.1			519.6 ± 51.1		
100~	820 (29.7)	3235.1 ± 395.7			523.9 ± 53.8		
≥200	133 (4.8)	3310.9 ± 394.9			530.1 ± 57.2		
Prepregnancy BMI			17.92^a^	<0.001		1.38^a^	0.248
Under weight (<18.5)	594 (21.5)	3095.4 ± 365.9			518.8 ± 46.7		
Normal weight (18.5~)	1768 (64.1)	3213.1 ± 404.9			522.8 ± 53.4		
Overweight (24~)	317 (11.5)	3260.3 ± 447.1			518.4 ± 49.6		
Obesity (≥28)	79 (2.9)	3234.8 ± 449.0			518.6 ± 53.5		
Maternal smoking			1.75^b^	0.080		0.34^b^	0.734
No	2754 (99.9)	3194.3 ± 406.7			521.3 ± 52.1		
Yes	4(0.1)	2837.5 ± 286.9			512.5 ± 25.0		
Passive smoking			0.55^b^	0.583		0.65^b^	0.518
No	1875 (68.0)	3196.7 ± 406.8			521.8 ± 53.7		
Yes	883 (32.0)	3187.6 ± 406.8			520.4 ± 48.3		
Alcohol consumption			−0.89^b^	0.373		−0.62^b^	0.539
No	2754 (99.8)	3193.5 ± 406.6			521.3 ± 52.0		
Yes	4 (0.2)	3375.0 ± 499.2			537.5 ± 47.9		
Adverse pregnant history			−2.43^b^	0.015		−0.12^b^	0.906
No	1601 (58.1)	3177.8 ± 400.7			521.2 ± 52.0		
Yes	1157 (41.9)	3215.9 + 413.9			521.5 ± 52.0		

^a^Denotes the *F* value.

^b^Denotes the value is the *t* value.

BMI, body mass index.

Universal correlation analyses showed that maternal Se exposure levels during the first trimester were negatively associated with birth weight (*r* = −0.04, *P* = 0.05), placental weight (*r* = −0.04, *P* = 0.05), and placental weight was positively associated with birth weight (*r* = 0.38, *P* < 0.001) (eFigure S3; http://links.lww.com/EE/A128). Similar results were observed among the maternal Se exposure levels in the third trimester, placental weight, and birth weight (eFigure S3; http://links.lww.com/EE/A128).

### Associations Between Prenatal Se Exposure and Birth Weight

Table 2 shows the adjusted associations between maternal exposures to Se during pregnancy and birth weight of neonates. We observed a negative association between maternal Se exposure during the first trimester with birth weight. Each IQR (0.53 μg/g creatinine) increment in the urine ln-Se concentration was associated with a mean 21.7 g (95% CI = −41.3g to −2.1g) decrease in birth weight after adjustment for potential covariates. Compared with the Q1 of ln-Se concentrations in urine, significant lower birth weight was also found in the Q4 (β =  −45.7g, 95% CI = −90.7g to −0.7g). The trend analysis was statistically significant (*P* = 0.018).

**Table 2.** Associations of maternal exposures to Se during pregnancy with birth weight and placental weight.

	Birth weight (g)	Placental weight (g)
ln-Se concentrations (μg/g creatinine) measured in early pregnancy (n = 2151)	β (95% CI)	β (95% CI)
Per IQR (0.53 μg/g creatinine) increase	−21.71 (−41.29 to −2.12)*	−3.60 (−6.28 to −0.93)**
Quartile 1 (≤3.40)	1	1
Quartile 2 (3.40–3.65)	18.37 (−26.26 to 63.00)	−1.51 (−7.61 to 4.58)
Quartile 3 (3.65–3.94)	−16.10 (−60.92 to 28.73)	−5.57 (−11.70 to 0.55)
Quartile 4 (≥3.94)	−45.72 (−90.71 to −0.73)*	−5.66 (−11.81 to 0.49)
P for trend	0.018*	0.034*
ln-Se concentrations (μg/g creatinine) measured in late pregnancy (n = 1383)		
Per IQR (0.63μg/g creatinine) increase	−24.46 (−43.19 to −5.73)*	−1.85 (−4.55 to 0.86)
Quartile 1 (≤3.36)	1	1
Quartile 2 (3.36–3.67)	43.47 (−11.97 to 98.91)	6.68 (−1.34 to 14.69)
Quartile 3 (3.67–3.99)	−31.84 (−87.35 to 23.67)	0.68 (−7.35 to 8.71)
Quartile 4 (≥3.99)	−30.31 (−86.38 to 25.76)	−1.87 (−9.98 to 6.24)
P for trend	0.063	0.361

Multiple-regression models adjusted for maternal age, family income, maternal education, gestation weeks, prepregnancy BMI, parity, gravidity, and newborn sex, maternal smoking, passive smoking, alcohol consumption, adverse pregnancy history.

*P < 0.05; **P < 0.01.

CI, confidential interval.

We also observed that each IQR (0.63 μg/g creatinine) increment in the urine ln-Se concentration in the third trimester was associated with a reduction of 24.5 g (95% CI = −43.2g to−5.7g) in birth weight. The pregnant women with the Q2 of ln-Se concentrations in urine had higher birth weight (β = 43.5g, 95% CI = −12.0g to 98.9g) than those with the Q1, but the Q3 (β = −31.8g, 95% CI = −87.4g to 23.7g) and Q4 (β = −30.3g, 95% CI = −86.4g to 25.8g) of ln-Se concentrations in urine had lower birth weight than those with the Q1 of ln-Se concentrations though without significance.

### Associations Between Maternal Exposures to Se and Placental Weight

We found significant negative associations of ln-Se concentrations in the first trimester with placental weight (Table 2). In the multivariable models, each IQR (0.53 μg/g creatinine) increase in urine ln-Se concentration was associated with a 3.6 g (95% CI = −6.3g to −0.9g) decrease in placental weight. The pregnant women with the Q2 (β = −1.5g, 95% CI = −7.6g to 4.6g), Q3 (β = −5.6g, 95% CI = −11.7g to 0.6g) and Q4 (β = −5.7g, 95% CI = −11.8g to 0.5g) of ln-Se concentrations in urine had lower placental weight than those with the Q1 of ln-Se concentrations, although the associations were not significant. But the trend analysis was statistically significant (*P* = 0.034). We did not find a significant association between maternal exposure to Se in the third trimester and placental weight.

### Mediation of Placental Weight Loss in the Association Between Maternal Se Exposure During Pregnancy and Birth Weight

**Figure 1.**
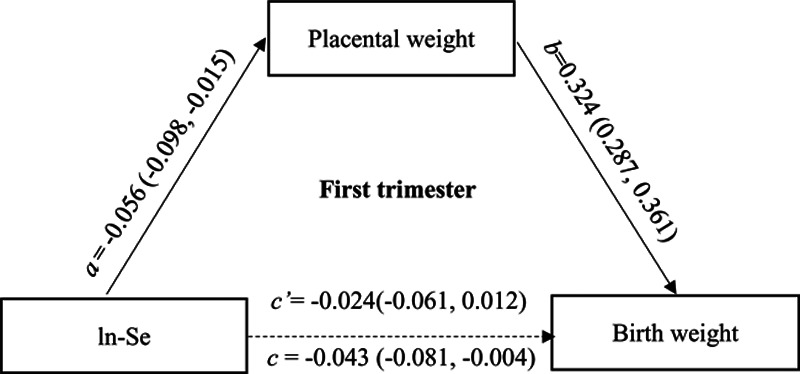
The mediation effects of placental weight change on the association between maternal Se exposures during the first trimester and birth weight. ln-Se: The urinary Se concentrations were corrected by creatinine and transformed by natural logarithm. Multiple-regression models adjusted for maternal age, family income, maternal education, gestation weeks, prepregnancy BMI, parity, gravidity, and newborn sex, maternal smoking, passive smoking, alcohol consumption, adverse pregnancy history.

**Figure 2.**
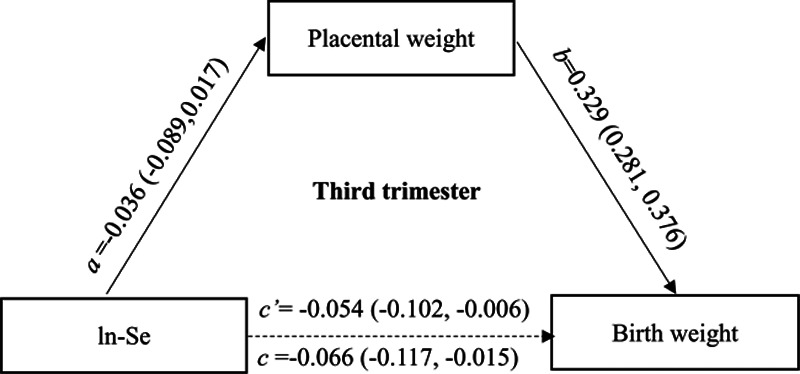
The mediation effects of placental weight change on the association between maternal Se exposures during the third trimester and birth weight. ln-Se: The urinary Se concentrations were corrected by creatinine and transformed by natural logarithm. Multiple-regression models adjusted for maternal age, family income, maternal education, gestation weeks, prepregnancy BMI, parity, gravidity, and newborn sex, maternal smoking, passive smoking, alcohol consumption, adverse pregnancy history.

We found that each gram increment in placental weight was associated with an average of 2.6 g (95% CI = 2.2g to 2.9g) increase in birth weight after adjustment for potential confounders. According to the definition of mediation effect, 44.2% of the effects of Se exposure measured in the first trimester on birth weight could be mediated by the change of placental weight (Figure 1), and 18.2% of the effects of Se exposure measured in the third trimester could be mediated by placental weight change (Figure 2).

## Discussion

The present study employed a prospective birth cohort study to investigate the associations of maternal Se exposure during pregnancy with birth weight and placental weight. We have observed that maternal Se exposure during the first and third trimesters was negatively associated with birthweight, and the reduction of placental weight may partially mediate the association between maternal Se exposure and birth weight loss. These findings extended our understanding of the effects of Se exposure on fetal development, have important implications to prevent maternal high exposure to Se during pregnancy, and to reduce the risk of low birthweight.

Seldom previous studies have investigated the effects of maternal Se exposure on birth weight, and their results were inconclusive. Studies in Poland and Spain have found an inverse relationship between maternal Se level and birth weight.^2,10^ A study in the Yangtze River estuarine zone of China also observed marginal significances in the negative associations between Se levels and birth weight.^4^ These results were consistent with our findings. However, positive associations between Se exposures and birth weight were also found in the United Kingdom,^29^ and in China.^26^

This heterogeneous result may be explained by the differences in study design, exposure assessment, study population, ethnics, and exposure levels. Out of these factors, differences in Se exposure level may play an important role. As we found in this study, low exposure to Se may benefit fetal development, but high Se exposure may result in adverse effects on fetal health. For example, in Sun et al’ study which found a positive association between Se exposure and birth weight, the Se exposure level in pregnant women was very low with a median level of 5.07 μg/g creatine in maternal urine.^26^ By contrast, the present study was conducted in Guangzhou, which is located on the southeast coast of China. The primary diets of the Guangzhou population are seafood,^30^ and it has been demonstrated that Se is abundantly present in marine fish.^3^ Therefore, Se exposure of participants in this study is much higher than in other studies. The mean Se concentrations in maternal urine during the first and third trimesters were 44.6 creatinine and 46.7 creatinine, respectively. This suggests that future studies are needed to identify the safety threshold of Se level in the human body, which is crucial to assess the potential embryotoxicity and fetotoxicity of Se.

We also observed the association of maternal Se exposure during pregnancy with placental weight loss, which is consistent with a study conducted in Poland.^10^ In contrast, another study has found a positive association between cord blood Se level and placental weight.^31^ The heterogeneity may also be related to the variation of exposure levels. For example, the mean Se exposure level (0.175 ± 0.021 μg/g) in placental tissues measured in Al-Saleh et al.’ study was much lower than that (>0.6 μg/g) in Zadrożna et al.’s study.^10,31^ Besides, some studies measured Se exposure in various tissues, including urine, umbilical cord blood, and placental tissues, which may also lead to variation in the association between Se exposure and placental weight. For example, it was suggested that maternal urine samples and umbilical cord blood samples showed some differences in response to Se exposure.^11^

We have also observed that the change of placental weight might partly mediate the association of maternal Se exposure on birthweight. Although we did not find a study that examined the mediation of placental weight change in the effects of Se exposure on birth weight, some studies found analogical results. For example, a study in the USA has observed placental weight was a mediator in the relation between placental Cd concentration and reduced birth weight, implicated that the role of interacting essential and contaminant elements on the birth weight that may be mediated by changes in the growth and function of the placenta.^16^ A case-control study identified that placental weight mediated the association between prenatal cooking oil fumes exposure and full-term low-birth weight.^32^ Another cohort study has implicated placenta might be one of the potential mediators of the association between prenatal nitrogen dioxide (NO_2_) exposure and birth weight.^19^ Furthermore, Niu et al. have found placenta plays an intermediary role in the association between maternal second-hand smoke exposure during pregnancy and full-term low-birth weight.^33^ These results suggest the important role of placental development in the adverse effects of environmental contamination on fetal health.

The biological mechanisms of the placenta mediating the association between maternal exposure to Se and birth weight loss are largely unknown. One possible mechanism is the oxidative stress reaction by decreasing the activity of cytochrome c oxidase (CCO).^10^ High level of Se could decrease the activity of CCO, which might lead to the hypoxia of the placenta and affect its normal function, and finally cause the reduction of birth weight.^34–36^ In addition, the increase of Se concentration may cause the decrease of Cu and Zn concentration in the human body and indirectly affect the production of superoxide dismutase (Cu–Zn SOD),^37^ which is closely interrelated to antioxidant functions of the placenta, and ultimately limits the growth of the fetus.^35,38^

### Strengths and Limitations

This study has several strengths. First, we have conducted a prospective birth cohort study which could provide more convincing evidence of causal argument for the association of maternal Se exposure and birth weight. Second, the study has multiple sources of detailed maternal and children’s information through questionnaire interviews, maternal medical records, and socioeconomic factors, which allowed us to sufficiently adjust for potential confounders. Third, the mediating role of placental weight change in the association between prenatal Se exposure and birth weight could add more information of understanding the causative chain of adverse birth outcomes.

One limitation of this study is that all of the subjects were recruited from one hospital, which may limit the generalization of our findings. In addition, the sample size of our study was not enough to determine the thresholds of maternal Se exposure affecting birth weight and placental weight. Third, we have excluded 1521 pregnant women without urine samples which may produce a potential selection bias. The characteristics of included and excluded participants were shown in eTable S2; http://links.lww.com/EE/A128. Fourth, we did not collect information on variables such as fish consumption and other nutrients such as PUFAs. As a result, we could not control for the confounding effects of these variables on our findings. In addition, we did not adjust for the impacts of other metal exposures, such as Hg and As, on the associations between Se and birth weight. It has been demonstrated that higher Hg exposure could also decrease birth weight,^31^ and there is an interactive effect between Se and As exposures on human health.^39^ Finally, urinary Se is an indicator of short-term exposure, and measured at one spot time might not accurately reflect long-term Se level throughout the whole pregnancy.^40,41^ Further studies are needed to explore the dynamic changes of metal elements which could observe the main effect time of Se on maternal and fetal health.

## Conclusions

This study provided the preliminary evidence that elevated maternal Se exposure during pregnancy was negatively associated with birth weight, the reduction of placental weight may partially mediate the association of prenatal Se exposure and birth weight. This finding extended our understanding of the adverse effects of Se exposure on fetal health and the potential pathway of effects. Therefore, pregnant women need to take measures to reduce Se exposure during pregnancy.

## Supplementary Material


