# Supplementary material for: The mediation effect of placental weight change in the association between prenatal exposure to selenium and birth weight: Evidence from a prospective birth cohort study in China
Source: Environ Epidemiol. 2021 Apr 2;5(2):e139. doi: 10.1097/EE9.0000000000000139 (PMC8043733; doi:10.1097/EE9.0000000000000139)
Supplement: Supplementary file 1 [file ee9-5-e139-s001.docx]

**Supplementary materials**

**The mediation effect of placental weight change in the association between prenatal exposure to selenium and birth weight: evidence from a prospective birth cohort study in China**

Jiaqi Wang^1,2#^, Rui Qian^3#^, Yiding Wang^4^, Moran Dong^1,5^, Xin Liu^1^, He Zhou^1,2^, Yufeng Ye^6^, Guimin Chen^1,5^, Dengzhou Chen^1,2^, Lixia Yuan^1,7^, Jianpeng Xiao^1^, Guanhao He^1^, Jianxiong Hu^1^, Weilin Zeng^1^, Zuhua Rong^1^, Qianqian Zhang^4^, Mengya Zhou^4^, Juan Jin^2^, Jingjie Fan^8^ , Jiufeng Sun^1^, Wenjun Ma^1,9^, Bo Zhang^4,5*^, Tao Liu^1,9^*

1.Guangdong Provincial Institute of Public Health, Guangdong Provincial Center for Disease Control and Prevention, Guangzhou, 511430, China

2.School of Public Health, Guangdong Pharmaceutical University, Guangzhou, 510080, China

3. Statistical Information Center for Health and Family Planning Bureau of Foshan, Foshan, 528000, China

4. Food Safety and Health Research Center, School of Public Health, Southern Medical University, Guangzhou, 510515, China

5. School of Public Health, Southern Medical University, Guangzhou, 510515, China

6. Guangzhou Panyu Central Hospital, Guangzhou, 511400, China

7. School of Public Health, Sun Yat-Sen University, Guangzhou, 510080, China

8. Department of Prevention and Health Care, Shenzhen Maternity ＆ Child Healthcare Hospital, Southern Medical University, Shenzhen, 518028, China

9. School of Medicine, Jinan University, Guangzhou, 510632, China

^#^ These authors contributed equally to this work.

* To whom correspondence should be addressed

Tel: +86-20-31051632, Fax: +86-20-31051652 (Tao Liu)

Tel: +86-20-61648324 Fax: +86-20-61648324 (Bo Zhang)

Mailing address:

No.160, Qunxian Road, Panyu District, Guangzhou, Guangdong, 511430, China (Tao Liu).

E-mail address: [gztt_2002@163.com](mailto:gztt_2002@163.com) (Tao Liu)

No.1023, South Shatai Road, Baiyun District, Guangzhou, Guangdong, 510515, China (Bo Zhang).

E-mail address: zhangbo2018@smu.edu.cn (Bo Zhang)

**Table of Contents**

Figure S1. Chart of the cohort study participants selection.

**Supplementary texts**: Mediation analysis

**Supplementary figures**

Figure S2. The framework of mediation analysis.

Figure S3. The correlations among birth weight, placental weight and maternal urinary selenium levels.

Panel A: The correlations among birth weight, placental weight and maternal urinary selenium levels in the first trimester.

Panel B: The correlations among birth weight, placental weight and maternal urinary selenium levels in the third trimester.

Ln-Se: The urinary Se concentrations were corrected by creatinine and transformed by natural logarithm.

*:P-value <0.05; **: P-value < 0.01, ***: P-value < 0.001.

**Supplementary tables**

Table S1 Se concentrations in participants with different characteristics.

Table S2 Differences in the characteristic of included and excluded participants.


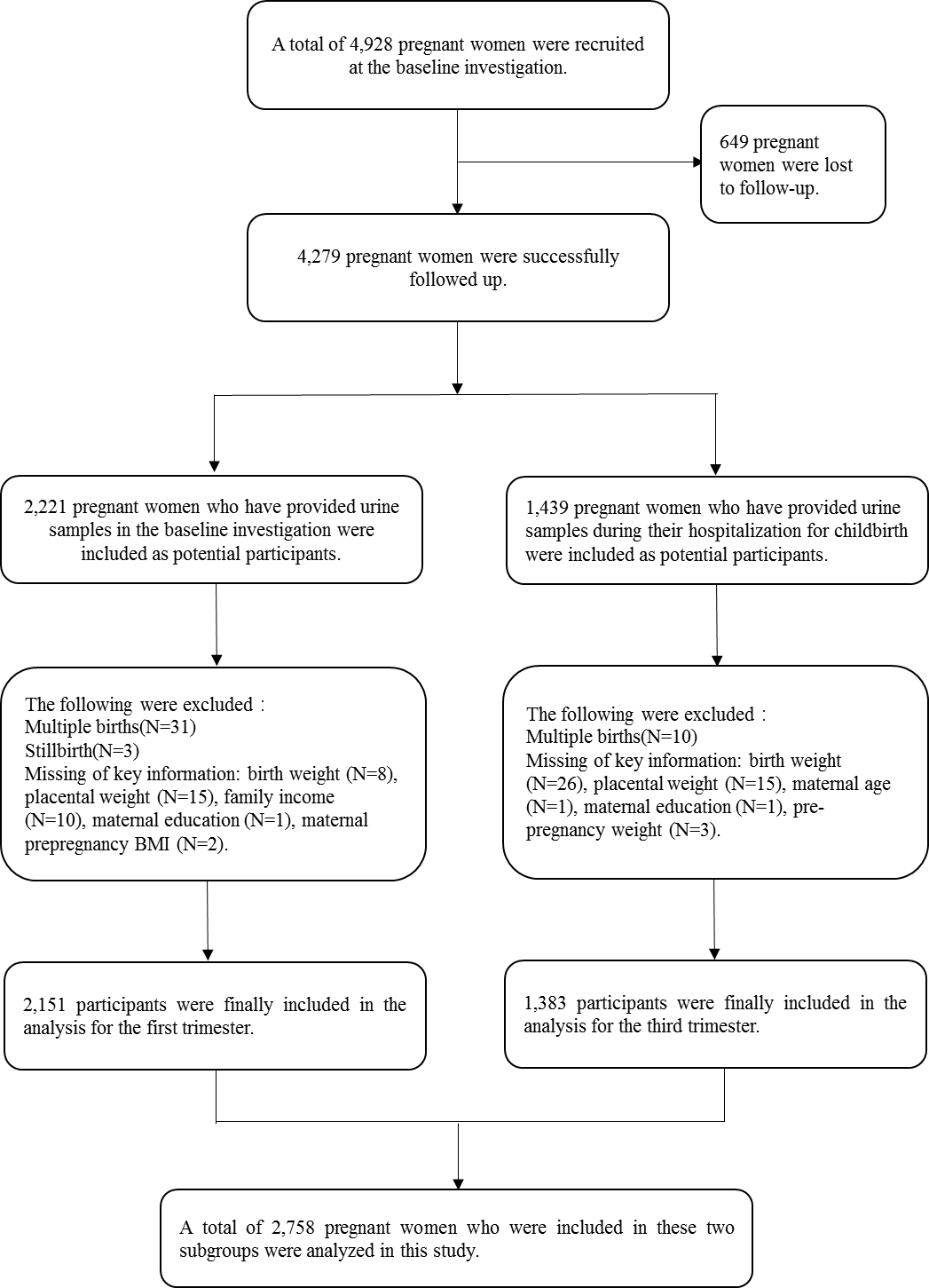


Figure S1. Chart of the cohort study participants selection.

**Supplementary texts**

**Mediation analysis**

The method illustrated in Mackinnon et al.’s study was employed to estimate the mediating effects of placental weight (Figure S2)^1^. According to the theory of mediation analysis, four steps are needed to analyze the mediation effect. First, a significant relation (coefficient: *c*) of the independent variable(X) to the dependent variable(Y) is required. Second, a significant relation(*a*) of X to the hypothesized mediating variable(M) is required. Third, M must be significantly related(*b*) to Y when both X and M are predictors of Y. Fourth, the coefficient (*c*) relating X to Y must be larger (in absolute value) than the coefficient (*c’*) relating X to Y in the regression model with both X and M predicting Y. These causal steps approach to assessing mediation has been the most widely used method to assess mediation. The equations can be described as:

$Y=i_{1}+cX+e_{1}$ (1)

$Y=i_{2}+c^{'}X+bM+e_{2}$ (2)

$M=i_{3}+aX+e_{3}$ (3)

*b*

*a*

X

S

Y

*c (c’)*

*(c’)*

M

Figure S2. The framework of mediation analysis

**References**

1. MacKinnon DP, Fairchild AJ, Fritz MS. Mediation analysis.*Annu Rev Psycho* 2007;58:593-614.


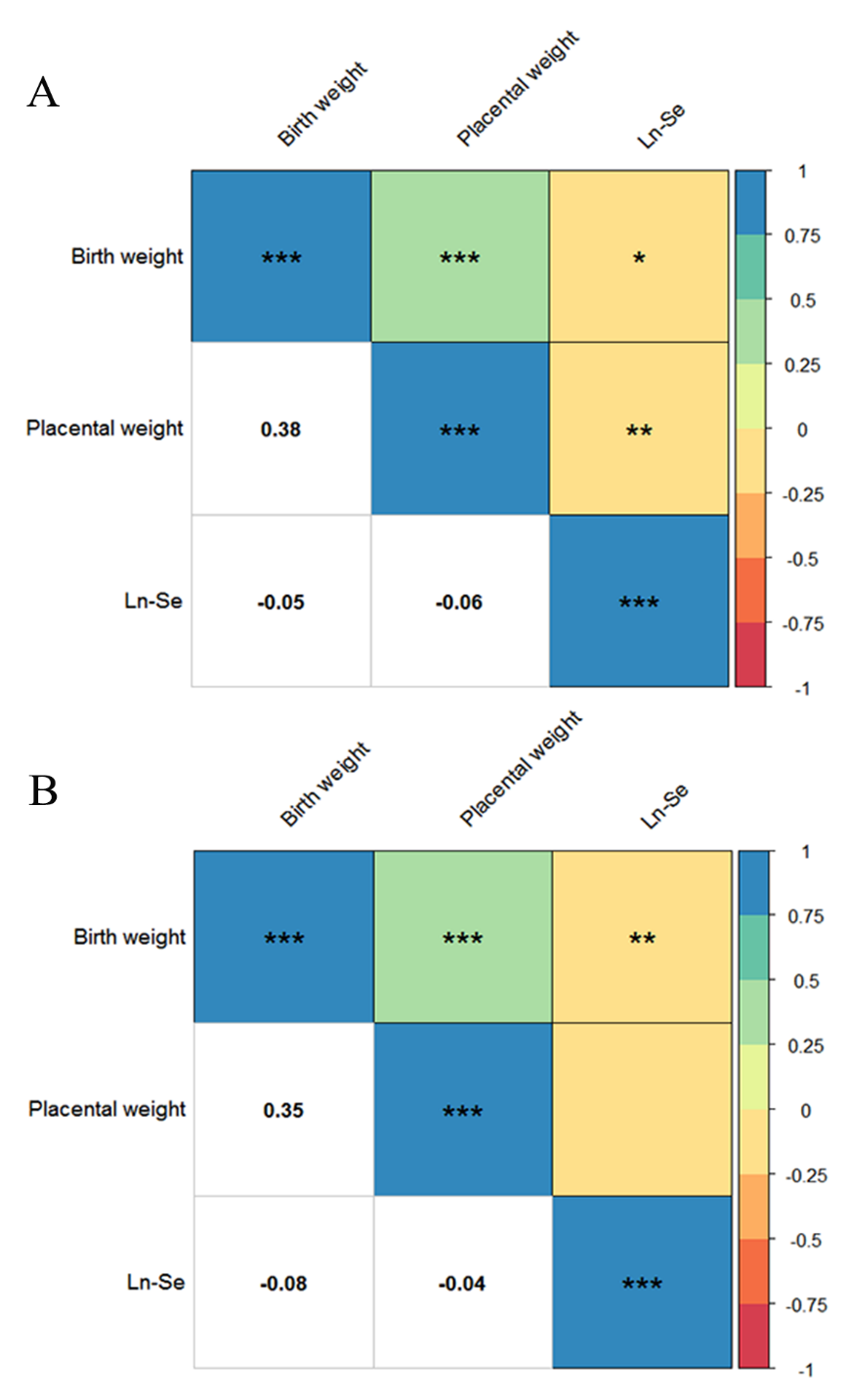


Figure S3. The correlations among birth weight, placental weight and maternal urinary selenium levels.

Panel A: The correlations among birth weight, placental weight and maternal urinary selenium levels in the first trimester.

Panel B: The correlations among birth weight, placental weight and maternal urinary selenium levels in the third trimester.

Ln-Se: The urinary Se concentrations were corrected by creatinine and transformed by natural logarithm.

*:P-value <0.05; **: P-value < 0.01, ***: P-value < 0.001.

**Table S1** Se concentrations in participants with different characteristics.

|  | Early pregnancy | | Late pregnancy | |
| --- | --- | --- | --- | --- |
|  | N (%) | Se concentrations (μg/g creatinine)  Mean ± SD | N (%) | Se concentrations (μg/g creatinine)  Mean ± SD |
| All | 2151(100) | 44.6±25.0 | 1383(100) | 46.7±31.8 |
| Maternal age(Years) |  |  |  |  |
| <25 | 107(5.0) | 44.1±20.0 | 75(5.4) | 44.9±29.0 |
| 26~29 | 704(32.7) | 43.5±22.7 | 489(35.4) | 45.6±27.2 |
| 30~34 | 667(31.0) | 42.8±20.7 | 448(32.4) | 46.8±26.5 |
| ≥35 | 673(31.3) | 47.7±31.1 | 371(26.8) | 48.4±42.4 |
| Gestational age (Weeks) |  |  |  |  |
| <37 | 95(4.4) | 46.9±26.0 | 16(1.2) | 43.3±27.7 |
| 37~41 | 1956(90.9) | 44.6±25.0 | 1285(92.9) | 46.9±28.9 |
| ≥42 | 100(4.6) | 43.2±25.3 | 82(5.9) | 37.0±15.2 |
| Infant sex |  |  |  |  |
| Male | 1159(53.9) | 45.1±26.4 | 746(54.0) | 45.7±35.2 |
| Female | 992(46.1) | 44.1±23.4 | 637(46.1) | 47.9±27.4 |
| Parity |  |  |  |  |
| 0 | 808(37.6) | 44.5±24.0 | 433(31.3) | 48.7±27.6 |
| 1 | 1316(61.2) | 44.8±25.8 | 838(60.6) | 47.3±.35.1 |
| ≥2 | 27(1.3) | 41.1±16.1 | 112(8.1) | 34.4±14.1 |
| Gravidity |  |  |  |  |
| 1 | 578(26.9) | 44.5±24.8 | 374(27.0) | 46.2±25.4 |
| 2 | 864(40.1) | 44.6±22.9 | 558(40.3) | 48.5±38.9 |
| 3 | 474(22.1) | 44.0±22.6 | 293(21.2) | 46.1±27.9 |
| ≥4 | 235(10.9) | 46.4±35.8 | 158(11.4) | 42.5±23.7 |
| Maternal education (Years) |  |  |  |  |
| ≤12 | 341(15.9) | 40.1±19.9 | 209(15.1) | 42.0±23.9 |
| 13~15 | 1226(57.0) | 45.1±26.2 | 792(57.3) | 47.2±34.8 |
| >15 | 584(27.2) | 46.3±25.1 | 382(27.6) | 48.2±29.1 |
| Household income (×1000Yuan) |  |  |  |  |
| <30 | 71(3.3) | 43.2±24.7 | 73(5.3) | 36.0±23.1 |
| 30~ | 1353(62.9) | 44.1±23.7 | 821(59.4) | 47.0±29.4 |
| 100~ | 624(29.0) | 45.5±25.6 | 421(30.4) | 47.6±37.0 |
| ≥200 | 103(4.8) | 46.9±36.2 | 68(4.9) | 48.5±33.3 |

Note: BMI: body mass index.

**Table S1** Se concentrations in participants with different characteristics (*continued*).

|  | Early pregnancy | | Late pregnancy | |
| --- | --- | --- | --- | --- |
|  | N (%) | Se concentrations (μg/g creatinine)  Mean ± SD | N (%) | Se concentrations (μg/g creatinine)  Mean ± SD |
| Pre-pregnancy BMI |  |  |  |  |
| Under weight (<18.5) | 464(21.6) | 47.3±32.6 | 296(21.4) | 46.8±28.2 |
| Normal weight (18.5~) | 1377(64.0) | 43.9±21.9 | 903(65.3) | 47.0±34.4 |
| Over weight (24~) | 246(11.4) | 44.1±25.6 | 151(10.9) | 45.0±24.7 |
| Obesity (≥28) | 64(3.0) | 43.0±20.9 | 33(2.4) | 45.6±20.4 |
| Passive smoking |  |  |  |  |
| No | 1431(66.5) | 44.3±25.5 | 940(68.0) | 46.0±33.5 |
| Yes | 720(33.5) | 45.3±24.1 | 443(32.0) | 48.2±28.2 |
| Adverse pregnant history |  |  |  |  |
| No | 1235(57.4) | 44.4±23.5 | 804(58.1) | 46.9±34.2 |
| Yes | 916(42.6) | 44.9+26.9 | 579(41.9) | 46.6±28.4 |

Note: BMI: body mass index.

**Table S2** Differences in the characteristic of included and excluded participants

|  | Excluded participants  (n, %) | Included participants  (n, %) | *χ*^2^ | *P* value |
| --- | --- | --- | --- | --- |
| Maternal age (Years) |  |  | 0.28 | 0.963 |
| <25 | 79(5.2) | 145(5.3) |  |  |
| 26~29 | 504(33.2) | 919(33.3) |  |  |
| 30~34 | 544(35.8) | 967(35.1) |  |  |
| ≥35 | 392(25.8) | 727(26.3) |  |  |
| Gestational age (Weeks) |  |  | 122.84 | <0.001 |
| <37 | 188(12.6) | 100(3.6) |  |  |
| 37~41 | 1243(83.1) | 2526(91.6) |  |  |
| ≥42 | 65(4.3) | 132(4.8) |  |  |
| Infant sex |  |  | 3.63 | 0.06 |
| Male | 767(51.0) | 1492(54.1) |  |  |
| Female | 737(49.0) | 1266(45.9) |  |  |
| Parity |  |  | 327.84 | <0.001 |
| 0 | 338(22.2) | 957(34.7) |  |  |
| 1 | 846(55.6) | 1671(60.6) |  |  |
| ≥2 | 337(22.2) | 130(4.7) |  |  |
| Gravidity |  |  | 25.26 | <0.001 |
| 1 | 524(34.5) | 754(27.3) |  |  |
| 2 | 529(34.8) | 1106(40.1) |  |  |
| 3 | 304(20.0) | 598(21.7) |  |  |
| ≥4 | 162(10.7) | 300(10.9) |  |  |
| Maternal education (Years) |  |  | 1.51 | 0.471 |
| ≤12 | 260(17.1) | 433(15.7) |  |  |
| 13~15 | 849(56-0) | 1575(57.1) |  |  |
| >15 | 408(26.9) | 750(27.2) |  |  |
| Household income (×1000Yuan) |  |  | 20.36 | <0.001 |
| <30 | 116(7.8) | 123(4.5) |  |  |
| 30~ | 872(58.4) | 1682(61.0) |  |  |
| 100~ | 429(28.8) | 820(29.7) |  |  |
| ≥200 | 75(5.0) | 133(4.8) |  |  |
| Pre-pregnancy BMI |  |  | 1.23 | 0.747 |
| Under weight (<18.5) | 318(21.0) | 594(21.5) |  |  |
| Normal weight (18.5~) | 961(63.5) | 1768(64.1) |  |  |
| Over weight (24~) | 191(12.6) | 317(11.5) |  |  |
| Obesity (≥28) | 43(2.9) | 79(2.9) |  |  |

Note: BMI: body mass index.
